# Pregnant and Homeless in the UK: A Qualitative Analysis of Maternal Experiences in Temporary Accommodation

**DOI:** 10.1111/birt.12919

**Published:** 2025-04-04

**Authors:** Sara Cumming, Andrew Symon

**Affiliations:** ^1^ Mother, Infant and Child Research Group School of Health Sciences University of Dundee Dundee Scotland

**Keywords:** access to health services, anxiety, homeless, maternal health, pregnant, qualitative research, temporary accommodation

## Abstract

**Background:**

In the UK, families in temporary accommodation reached record numbers in 2023. Pregnant mothers experiencing homelessness are at risk of poor health outcomes, yet little is known about their experiences. Most biomedical research emphasizes obstetric outcomes rather than maternal experiences. Our study aimed to explore maternal experiences of pregnancy while living in temporary accommodation in the UK.

**Methods:**

Using an interpretivist paradigm and critical feminist theory, we collected and analyzed semi‐structured interview narratives from pregnant and postnatal mothers experiencing homelessness. Interviews with key workers from relevant Third Sector Organisations provided complementary insights. Study planning included Patient and Public Involvement. Data were analyzed using reflexive thematic analysis.

**Results:**

Fourteen mothers and six keyworkers were interviewed. Reflexive thematic analysis generated three themes. Theme one, *Pregnant/postnatal bodies in unsafe spaces*, described participants' experiences with unsafe accommodations, exposure to environmental hazards, and frequent moves which affected physical and mental health. In *Undermining mothers*, participants explained how the constant struggle to meet basic needs eroded opportunities to engage with caring roles and destabilized their sense of being “good” mothers. Together these contributed to pregnancy disengagement and feeling unprepared for birth. The third key theme, *Feeling unseen in midwifery blind spots*, describes barriers to accessing maternity services, as well as interactions with midwives that often reinforced feelings of being invisible.

**Discussion:**

Living in temporary accommodations whilst pregnant negatively impacts physical, mental, and emotional well‐being. Improving care for pregnant mothers experiencing homelessness requires systemic change within housing and maternity services to acknowledge housing security as an essential need for pregnant and parenting mothers.

## Graphical abstract

A co‐produced infographic summarises the experiences of mothers experiencing homelessness in pregnancy in the UK: feeling unsafe, feeling undermined as mothers and feeling unseen in midwifery blind spots. The full infographic is available at: https://doi.org/10.20933/100001309

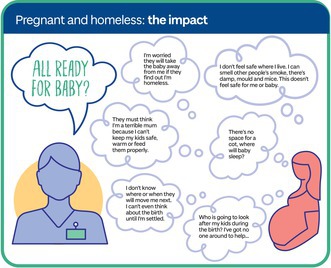

## 1 Introduction

Homelessness, a global phenomenon affecting millions, is increasingly recognized as a gendered experience [1]. Women have different pathways in and out of homelessness and experience different health challenges from being homeless [2]. Domestic violence [3] and relationship breakdown [2] are understood as common reasons women become homeless. However, wider systemic inequalities of poverty [4], financial insecurity [5], and expected social norms of caring roles [6] are also recognized as gender‐based drivers [2] of homelessness.

Although women constitute approximately 35% of US [7] and 38% of European [8] homeless populations, they are considered “largely invisible” [1, 5, 9, 10]. Images of homeless male street sleepers dominate public perception, policy and even research [10]. Furthermore, women represent a higher proportion of “hidden homeless” [10, 11] who sofa surf with friends or family and are often not counted in official statistics [10, 11, 12].

In the UK, being homeless is recognized as more than just being “roofless” [12]. Homelessness exists on a housing continuum [2, 13], including street sleeping, sofa surfing, and temporary accommodation (TA). Homeless families can spend years in TA [14]; women with children spend almost 5 months longer in TA than single, male, homeless applicants [15].

A survey by the Royal College of Midwives [16] (RCM) revealed most midwives have provided care for pregnant mothers experiencing homelessness. However, accurate numbers are unknown because of inconsistent data recording. Pregnant mothers who are homeless are included in homeless family statistics or sometimes counted as single until after birth [13, 17]. Across the UK, homeless families living in TA have increased since the COVID‐19 pandemic [18]. In March of 2023, the highest rates since records began were reported, with 104,510 households in England [19] and 15,039 in Scotland [15] living in TA.

Homelessness in pregnancy has been associated with low birth weight [7, 20], preterm birth [20, 21] and increased neonatal intensive care unit admissions [21]. However, most research has approached homelessness from a biomedical stance, emphasizing fetal outcomes [22, 23] and further rendering subjective maternal experiences invisible [13].

We sought to redress this dearth of women‐centered homelessness research by conducting a qualitative study with pregnant and recently postnatal mothers and keyworkers from Third Sector Organisations (TSOs). Our study was shaped by the overarching lens of critical feminist theory, which argues that gender cannot be understood in isolation from power imbalances, social structures, and intersecting forms of oppression [6, 24]. Our study explored homelessness in pregnancy at the intersections of systemic gendered and structural inequalities.

## 2 Methods

An interpretivist paradigm [25] underpinned the study's qualitative approach, and semi‐structured interviews were employed to collect and examine pregnancy experiences during homelessness. A small group of mothers with lived experiences of homelessness in pregnancy formed a Patient and Public Involvement (PPI) group to help shape the study.

A purposive sampling framework was co‐developed with the PPI group to ensure diversity in participant ethnicity, parity, and region. Eligible participants were aged over 16, between 28 weeks pregnant and 24 months postnatal, and identified as homeless during pregnancy. The definition of homelessness did not require participants to have homeless applications with local authorities, as we wanted to include hidden forms of homelessness.

Pregnant mothers with known Child Protection Orders to remove the baby at birth were not included for ethical reasons as some interview questions focused on parenting and feeding intentions. We also interviewed keyworkers aged 18 and over who were working in a TSO supporting women experiencing homelessness. Keyworker interviews were intended to access a wider range of experiences of homelessness in pregnancy to add complementary insight to participant accounts. Prior to active recruitment, the lead researcher (SC) used snowball sampling to identify contacts with key UK TSOs who support homeless families. Overall, five TSOs from London, South England, Northwest England and two cities in Scotland engaged. These TSOs gave eligible participants the Participant Information Sheet (PIS) and completed a ‘permission to refer’ form that allowed contact details and preferred language to be shared with the lead researcher. Contact was made after 24 h to allow participants time to consider before agreeing to interview. All participants received a small shopping voucher, in keeping with best practices [26], and were informed withdrawal would not revoke their voucher.

Due to the lack of existing literature, the interview topic guide (Data S1) was exploratory in approach. It was co‐produced by the lead researcher (SC) and the PPI group. A trauma‐informed research colleague reviewed it for sensitivity. Participants could choose the interview location, and all interviews were audio recorded with consent, transcribed verbatim, and anonymized. Participants were coded as W (woman) or K (keyworker) in numerical order.

Data analysis used reflexive thematic analysis [25] with an inductive approach. Continual researcher reflexivity using Braun and Clarke's [25] six steps helped maintain awareness of researcher positionality and the potential for subjective bias [27]. The co‐researcher (AS) randomly coded transcripts separately to further increase rigor [27]. Codes and themes were co‐generated through ongoing collaborative discussion. Recruitment was considered complete once the sampling framework was met and the richness of data collected was sufficient to saturate themes and theoretical concepts [28]. Critical feminist theory [6, 24] shaped the analysis in that we intentionally explored intersecting systemic and structural inequalities and power differentials affecting participant's experiences.

## 3 Results

### 3.1 Participants

Between December 2022 and March 2023, fourteen mothers and six keyworkers consented to be interviewed. Table 1 shows mothers' socio‐demographic details only. Demographic data were not collected for keyworkers. Interviews lasted between 34 and 107 min, and participants chose either face‐to‐face (8), telephone (6) or online (6) interviews. Three interviews with mothers used interpreters.

**TABLE 1:** Women's socio‐demographic information.

Age	Number (*N* = 14)
19–24	3
25–29	0
30–35	5
36–40	4
Not disclosed	2
Parity	
Primiparous	5
Multiparous (P2–P6)	9
Antenatal/postnatal	
AN: < 28 weeks^a^	1
AN: > 28 weeks	1
PN: 0–6 months	6
PN: 7–12 months	3
PN: 13–18 months	2
PN: 19–24 months	1
Ethnicity (self‐identified)	
White British	2
White Scottish	2
Gypsy Traveler	1
Black African	4
Eastern European	1
Bedoon	1
Pakistani	1
Bangladeshi	1
Not disclosed	1

^a^ Participant was also 12 months postnatal and therefore included.

Homeless experiences were diverse: street sleeping, sofa surfing, unlicensed houses of multiple occupancy, shelters, hostels, hotels, and council homeless accommodation. Homeless length ranged from 8 months to 8 years, and three mothers experienced subsequent pregnancies in TA. Mothers reported diverse causes of homelessness ranging from seeking asylum (*n* = 9), escaping gender‐based violence (*n* = 2), relationship breakdown (*n* = 1), eviction (*n* = 1) and financial insecurity (*n* = 1).

### 3.2 Findings

Using reflexive thematic analysis, we generated 21 codes that formed three main themes. These are explored below, illustrated with participant quotes. Quotes have been shortened for reading ease using “…” but the fundamental meaning is retained and the precise words used in speaking remain to reflect authenticity.

#### 3.2.1 Theme 1: Pregnant/Postnatal Bodies in Unsafe Spaces

All mothers reported feeling unsafe because of poor housing standards in TA. Common experiences included overcrowding, vermin, and exposure to damp and mold in sleeping areas. Health concerns centered around worrying about what impact these environmental hazards had on their pregnancy.

It was all pretty dangerous, there was holes in the walls and the flooring was broken. Once we'd moved in, we realised there was mice everywhere; there was mice in the children's beds and cupboards…It wasn't hygienic at all, there was like damp and mould, and it was just really scary living there. (W14's experience at 38 weeks pregnant)

Other residents were discussed as sources of threat from exposure to passive smoking (tobacco and drugs) in communal hallways, to hearing fights, and experiencing harassment.

A woman… was threatening to sexually, like, assault someone's kid…it was making me really scared because I was a lot more emotional as well during pregnancy…I felt I couldn't physically protect myself because I've got that bump to protect. (W2's experience when four months pregnant)

All keyworkers agreed that pregnancy increased feelings of physical vulnerability in TA. K1 explained that this was worse in mixed‐gender accommodation as pregnant mothers shared bathrooms with unknown male residents.

Women in hostel[s] have said, ‘I can't go to the bathroom because I know that there's people and men outside my door. I can smell the smoke. I can smell the alcohol’. Even if the bathroom is quite close, they're too scared to actually go out into the corridor. (K1)

Feeling unsafe left all mothers self‐isolating in small, overcrowded rooms. Described as ‘living in a cage’ by W6, isolation was exacerbated by strict rules restricting visitors which prevented crucial social support. W2 was placed in singles accommodation that meant her partner couldn't visit and increased her isolation.

Feeling isolated in TA also impacted perinatal mental health. All participants felt that living in TA caused stress, anxiety, and depression and four mothers with traumatic histories experienced suicidal thoughts. Further isolation was caused by frequent moves, often with only 24 h notice, that separated them from social support networks. All mothers were moved at least once in pregnancy (once = 6, twice = 3, three time = 2 and six times = 3). Two mothers were moved 3 weeks before giving birth, causing physical strain and stress. W13 moved five times in under 2 months, whilst W8 described being ‘shifted to different hotels’ weekly when she was 5 months pregnant. When W14 tried to explain to housing officers that she needed more time because she was heavily pregnant and had a toddler, she encountered inflexible rules.

I [was] eight months pregnant and they had given me 24 hours' notice to be out the property. It puts a lot of pressure on you, especially when you're so far on in your pregnancy… The council officers, they don't actually care, they just want you out of the property… it was like soul destroying. (W14)

#### 3.2.2 Theme 2: Undermining Mothers

Lack of access to cooking facilities in TA was common. Some hotels had no access to food outside set mealtimes; others only had a kettle in bedrooms or shared kitchens with multiple residents that made access difficult. Mothers reported eating cold, tinned foods or take‐aways throughout their pregnancies.

I lived off pot noodles and tinned pineapple… but that ended up making me really sick. (W2's experience when 4 months pregnant)

I would only eat twice a day, sometimes only bread. It's very difficult to have been pregnant in a hotel. When you're hungry overnight and you're not able to eat, I would feel my baby was hungry too. (W3 when 28 weeks pregnant)

Mothers reported worrying that poor nutrition in pregnancy would directly affect their unborn baby, while two attributed their baby's low birthweight to this.

I was eating a little but not that much. That's why, when I have the baby, she is very small; she was so tiny. (W10)

Regular food insecurity meant mothers could not follow healthy eating advice in pregnancy, or cook nutritional meals for older children, which left them feeling demoralized and undermined as mothers.

It just makes you feel really low… it made me feel like a bad parent because I couldn't provide for my child. (W14's experience when 8 months pregnant)

Both mothers and keyworkers reported that the regular food insecurity and constantly feeling unsafe were highly stressful and impacted pregnancy engagement, bonding, and preparation for birth.

They're dealing with the complexity of trying to remain safe in a place that is unfamiliar, in surroundings which are not constant, where they could be moved at short notice. So the preparation for baby, sometimes that's the last thing on a woman's mind. They aren't able to focus in on that emotional connection or bonding with that baby. (K3)

W9 recalled that her ‘mind was not there at all’ whilst W13 and W14 felt the constant worry of not knowing where they would be living postnatally, prevented them from bonding during pregnancy.

I was so stressed over what was going on, I couldn't think about anything else…Like my whole pregnancy, I couldn't enjoy it. I couldn't enjoy that he was gonna be born…Every day I wanted it to be a bit longer…I wanted to be settled somewhere in a permanent accommodation before baby was born so I knew that we were secure. (W15's experience when 34 weeks pregnant)

#### 3.2.3 Theme 3: Feeling Unseen in Midwifery Blind Spots

As a result of the challenges described in themes one and two, mothers entered maternity services already feeling unseen and undermined as mothers before they had given birth. Ten mothers described midwifery blind spots in services and during interactions with midwives.

### 3.3 Service Level Blind Spots

All mothers described barriers to accessing maternity care while experiencing homelessness. Moving frequently involved registering with new services in different health boards and delayed midwifery appointments, scans, and screening tests.

I weren't able to get hold of medication. I weren't able to see midwives… by the time you register or got an appointment they were saying I had to move again. You've gotta have an address… and you can't prove where you're living when you're living week to week in hotels. (W13's experience when 24 weeks pregnant)

Keyworkers reported digital exclusion was common with no internet access in most emergency TA. This was crucial for W2, who was unable to access key information about her pregnancy and birth choices in her digital maternity notes.

Nine mothers were seeking asylum and experienced increased barriers to accessing maternity care. All nine experienced increased financial difficulties, meaning they sometimes missed maternity appointments because they had already spent their limited funds on traveling to legal appointments or buying food. Two mothers had no recourse to public funds and declined obstetric tests because of escalating costs. Three delayed accessing antenatal care, one until 7 months pregnant, because they had been told by others that their baby would be removed if social services found out they were homeless and claiming asylum. K1 and K2 worked primarily with migrant mothers and confirmed these experiences of intersecting disadvantages. They felt lower levels of social support also made these mothers more vulnerable to further isolation when they were moved.

### 3.4 Midwifery Interactions

Three mothers reported direct experiences of racism or feeling judged by midwives.

The midwife, because of the way she was judging me, I wasn't able to talk with her when I go there. I just do my things and I go back. (W10)

There's nothing worse than going into a midwife appointment and explaining all this and them thinking that you can't cope, or you are not doing your best or you're putting your baby in danger. (W13)

In addition, ten mothers reported feeling that the midwife simply seemed ‘too busy’ (W8) to really ask how they were. Midwifery interactions were described as focusing on fetal well‐being almost exclusively, leaving mothers feeling further unseen. They explained that midwives did not acknowledge or engage with their homelessness. One mother recalled that her midwife refused to write a letter to the local council about her housing situation because it was ‘not her department’. (W8).

All participants discussed feeling unseen increased when midwives gave generic health advice that was unachievable in TA. Two mothers with gestational diabetes were told to walk after meals, despite telling midwives they did not feel safe leaving their rooms. Three others were given generic safe sleeping advice despite explaining they had no space for a cot or were bed‐sharing with several children and a newborn due to overcrowding.

You can't actually do a lot of the things they're advising you to do. (W14)

Conversely, four mothers who had been under specialist midwifery care described situations of feeling seen when care was adapted to their individual needs. Continuity of care also offered longer relational support throughout their pregnancy.

They would always go to my social worker meetings, and they made me feel like I was doing a good job…they never, ever made me feel like I would be less capable of being a mother because of being homeless. (W2)

## 4 Discussion

We believe this is the first UK qualitative study to specifically explore maternal experiences of pregnancy whilst living in TA. Our study was grounded in critical feminist theory [6, 24], allowing homelessness to be explored with attention to intersecting and systemic inequalities of poverty, race, and gender. Our findings highlight the ways homeless services and housing systems failed to see and respond to women's increased need to feel safe in pregnancy, which left them feeling undermined, destabilized, and demoralized as mothers. Midwifery blind spots often further exacerbated these feelings of being unseen.

Our findings concur with previous literature revealing how mothers often experience homeless accommodations as places of fear [4, 29]. Reports have identified that families are often placed alongside single residents, and children can witness substance use and violent behaviors [30]. Poor housing standards [31], overcrowding [14], and bed‐sharing [18] have also been reported alongside regular food insecurity from lack of access to cooking facilities [4, 14, 17, 18].

However, our study findings add further insight into how life in TA affects pregnancy experiences. Participants worried their physical health in pregnancy was affected by exposure to environmental hazards like mold, damp, vermin, and passive smoking. Inadequate cooking facilities left them further worried about reduced nutritional intake in pregnancy. While some research reports low levels of health literacy among pregnant mothers experiencing homelessness [23], the mothers in this study generally demonstrated high levels of health literacy. Neural tube defects, anemia, and low birth weight [32] are known consequences of poor nutrition in pregnancy, and maternity services actively disseminate this information to pregnant mothers to encourage healthy behaviors [33]. Participants felt undermined as mothers when they were constantly unable to eat nutritious food but were surrounded by information on the importance of healthy eating in pregnancy.

Bimpson et al.'s [17] study identified feelings of reduced parenting capacity amongst mothers living in TA with older or removed children. However, our study offers further insight as to how living in TA with constant fear, uncertainty, and food insecurity, not only destabilized and undermined their sense of being a ‘good’ mother but also impacted pregnancy engagement. The constant stress of trying to meet basic daily needs in TA and never knowing when or where they would be moved next, reduced participants' ability to engage with the pregnancy and to prepare for birth.

Participants reported that their worries about the health impacts of TA in pregnancy were largely unacknowledged and that their basic needs went unmet in housing systems and services. Homeless service design and government policies have been criticized for being gender‐blind [34] and failing to see or meet the needs of women experiencing homelessness. Critical feminists have argued this is rooted in gendered normative assumptions of women as homemakers that mark women experiencing homelessness as ‘deviant’ [11]. Services are therefore primarily designed for male street sleepers and there is a significant lack of family‐focused TA [35]. Despite pregnant mothers being given priority around housing needs [17], there are no national recommendations for TA standards specifically for families and pregnant mothers [30]. This means the health needs of pregnant mothers, and the health impacts of long‐term living in TA, remain largely invisible.

Participants' experiences of maternity care often reinforced these feelings of being unseen by systems that eroded their opportunities to engage in expected caring behaviors as “good” mothers. Responding to Ashley et al.'s [36] call to develop Critical Midwifery Studies and to reflect on midwifery's role in (re)producing injustice, our study also highlighted how these systemic inequalities were often reinforced through difficulties in accessing maternity care. Proof of address is not a legal requirement for registration with General Practitioner or maternity services [37], yet participants reported this was still an issue in practice. These barriers were increased for the mothers seeking asylum in our study; their experiences of pregnancy and homelessness were further shaped by intersecting identities tied to asylum‐seeking status, ethnicity and language which racialized healthcare encounters for some. Barriers to care amongst ethnic minority communities, and those seeking asylum, are increasingly reported [38, 39] but further research with pregnant minoritized communities is needed to understand their specific experiences of complex, multi‐factorial, structural exclusion and inequality whilst experiencing homelessness.

Interactions with midwives became critical moments where barriers and feelings of invisibility were often exacerbated. Ashley et al. [36] also call for critical reflection on the role of the midwife in maintaining systems of exclusion and discrimination that contribute to health inequalities. However, the RCM report ‘Supporting Midwives’ [40] recognizes midwifery care for women with multiple disadvantages depends on resources, staffing and capacity for professional autonomy. The report [40] recognizes that midwives need systemic changes in service commissioning that grants them the autonomy and flexibility to offer longer appointments for disadvantaged women. Our finding that midwives were seen as ‘too busy’ (W8) reflects perceptions of chronic understaffing in maternity services [41]. The Birthrights study [42] also identifies a clear need for women experiencing multiple disadvantages to be able to access specialist midwives, continuity of care and longer appointments. Only four mothers in the study had specialist midwifery care, reflecting the inadequacy of its provision across the UK.

The RCM report [40] also identified that midwives need more support to care for women made vulnerable by multiple systems of oppression. RCM guidance [37] advises asking direct questions about housing insecurity four times over the course of the pregnancy. This recommendation aligns with what participants shared with us; mothers wanted midwives to ‘lift the lid’ (K3) and ask directly about living conditions. Furthermore, overcrowding and lack of space for a cot can result in unsafe co‐sleeping practices [14]. Recent research [18, 43] has shown the need for health professionals to adapt safe sleeping advice in temporary accommodation to reduce the risk of Sudden Unexpected Death in Infancy. However, these RCM recommendations [37] have not yet been implemented by the NHS, and midwives continue to report a need for better training and education around supporting women with multiple disadvantages [37, 40].

Clinical improvements for pregnant mothers experiencing homelessness must start with awareness of these systemic inequalities shaping their pregnancy. Our concluding recommendations for change are centered around the study findings of feeling unseen, feeling undermined as mothers, and experiencing midwifery blind spots.

Firstly, there is a clear need for increased visibility of pregnant mothers within UK homeless data. NHS health boards need to audit the numbers of pregnant mothers experiencing homelessness within their services to counter this invisibility. Longitudinal research that follows health and birth outcomes of pregnant mothers experiencing homelessness is needed to understand the long‐term health impacts for pregnant mothers in TA. Research amongst minoritized pregnant communities experiencing homelessness is also needed.

Secondly, empowering midwives to offer longer appointments and have open conversations about homelessness needs to be supported at the service level [40]. Commissioned hours need to be increased so midwives have the professional autonomy and capacity to offer longer appointments to mothers experiencing homelessness. But simply discussing these needs will not ameliorate them: midwives also need to be supported with training, education, and resources to respond to these needs.

Finally, we join the increasing call from the Third Sector to develop homeless accommodation specifically for families and pregnant mothers [30, 35]. These services need to be designed for and by the families that use them to overcome the gender blindness of women's homelessness in the UK.

### 4.1 Strengths and Limitations

Our study offers insight into a rarely accessed population, and the qualitative approach allowed for a nuanced exploration of subjective experiences of homelessness during and after pregnancy. The sampling framework and range of TSOs ensured diverse voices were included. However, study limitations include a lack of gender diversity; we have used the term mother because that is how participants referred to themselves.

Our use of interpreters was a strength in widening inclusion but can also be a limitation as subtle, complex meanings can be lost in translation. Six interviews were conducted over the telephone, which may have limited the depth of content shared. Resource limitations meant the views of midwives were not sought, and the study would benefit from further research to gain this complementary viewpoint.

## 5 Conclusion

Our study revealed pregnant mothers living in TA in the UK are a relatively invisible and under‐examined population who experience systemic inequalities. The gender‐blindness of homeless services, policy, and research left participants feeling their increased need for a safe, secure home in pregnancy was largely unseen. Furthermore, experiences of food insecurity contributed to participants feeling destabilized and undermined as mothers. Living with constant safety fears, food insecurity, and housing uncertainty further undermined their ability to engage with their pregnancy and to prepare for birth. Maternity service barriers and midwifery care interactions presented critical moments where these inequalities and disadvantages could have been ameliorated; yet, more often, invisibility and frustration with harmful systems were exacerbated. Improving care for pregnant mothers experiencing homelessness depends on systemic change within homeless service provision and maternity care: increased professional midwifery autonomy that enables respectful, responsive, and individualized care delivered during longer appointments, as well as improved access to the basic material resources needed to birth and parent in safety and with confidence.

## Conflicts of Interest

The authors declare no conflicts of interest.

## Supporting information


Data S1.

## Data Availability

The data that support the findings of this study are available from the corresponding author upon reasonable request.
