# Supplementary material for: Pregnant and Homeless in the UK: A Qualitative Analysis of Maternal Experiences in Temporary Accommodation
Source: Birth. 2025 Apr 4;52(3):503–10. doi: 10.1111/birt.12919 (PMC12434225; doi:10.1111/birt.12919)
Supplement: Supplementary file 1 — Data S1. [file BIRT-52-503-s001.docx]

**Pregnant/Postnatal Person Interview Topic Guide**

**Primary Research Question:** Exploring experiences of pregnancy whilst homeless in temporary accommodation

1. **Pregnancy**

- Demographics: Age, Parity, Ethnicity, how long homeless/TA?
- How many weeks pregnant/postnatal are you now?
- Can you tell me where were you living when you found out you were pregnant?

1. **Housing**

- Can you tell me about where you are living just now?
- Can you tell me about any other places you have lived during this pregnancy?
- Can you tell me about what access to facilities you have had/not had?
- What is/was it like to be pregnant in that space?
- How do you think the experience of being homeless affects pregnancy?

1. **House and Home**

- What does it mean to you to have a home?

1. **Infant feeding**

- How are you planning to feed baby?
- What, if any, impact does experiencing homelessness have on your infant feeding choices and experiences?

1. **Maternity Services (Barriers and facilitators to care and access issues)**

- What has your experience of midwifery care been like in this pregnancy?
- What, if any, are the barriers in accessing maternity services for pregnant people experiencing homelessness?
- What do you think are the most important things midwives need to understand in order to support pregnant people in temporary accommodation?
- What do you think are your key needs in your midwifery care?

1. **Prioritising Participants Voice**

- Do you think there is anything else that should be asked of you, or you would like to talk about, that you think is important to know about experiencing homelessness in pregnancy?

**Keyworker Interview Topic Guide**

**Primary Research Question:** Exploring experiences of pregnancy whilst homeless in temporary accommodation

**Interview Guide**

1. **Work and experience**

- Can you give me a brief overview of your role and area of work just now
- How often do you encounter pregnant people in TA/homeless in this role?

1. **Impact**

- Based on experiences of pregnant women you have encountered in your work, how do you think living in temporary accommodation affects pregnancy?
- Do you think it influences their infant feeding choices?
- Can you tell me about any examples of infant feeding experiences that have been affected by living in temporary accommodation?

1. **Housing**

- Can you tell me about the types of temporary accommodation you have seen pregnant people living in?

1. **Maternity Service Interactions**

- Can you tell me about what kind of interactions, if any, do you have with midwives or maternity systems whilst working in your role supporting pregnant people experiencing homelessness?
- What, if any, are the barriers in accessing maternity services for pregnant people experiencing homelessness?
- What key things do you think midwives need to understand to improve support for pregnant people experiencing homelessness?

1. **Prioritising Participants Voice**

- Do you think there is anything else that should be asked of you, or is there anything else you think is important to discuss?
